# Symbiosis Comes of Age at the 10th Biennial Meeting of *Wolbachia* Researchers

**DOI:** 10.1128/AEM.03071-18

**Published:** 2019-04-04

**Authors:** Irene L. G. Newton, Barton E. Slatko

**Affiliations:** a Department of Biology, Indiana University, Bloomington, Indiana, USA; b Molecular Parasitology Group, New England BioLabs, Ipswich, Massachusetts, USA; University of Queensland

**Keywords:** *Wolbachia*, conference, symbiosis

## Abstract

Wolbachia pipientis is an alphaproteobacterial obligate intracellular microbe and arguably the most successful infection on our planet, colonizing 40% to 60% of insect species. Wolbachia spp. are also present in most, but not all, filarial nematodes, where they are obligate mutualists and are the targets for antifilarial drug discovery. Although Wolbachia spp. are related to important human pathogens, they do not infect mammals but instead are well known for their reproductive manipulations of insect populations, inducing the following phenotypes: male killing, feminization, parthenogenesis induction, and cytoplasmic incompatibility (CI). The most common of these, CI, results in a sperm-egg incompatibility and increases the relative fecundity of infected females in a population. In the last decade, Wolbachia spp. have also been shown to provide a benefit to insects, where the infection can inhibit RNA virus replication within the host. Wolbachia spp. cannot be cultivated outside host cells, and no genetic tools are available in the symbiont, limiting approaches available for their study. This means that many questions fundamental to our understanding of Wolbachia basic biology remained unknown for decades. The 10th biennial international Wolbachia conference, Wolbachia Evolution, Ecology, Genomics and Cell Biology: A Chronicle of the Most Ubiquitous Symbiont, was held on 17 to 22 June 2018 in Salem, MA. In this review, we highlight the new science presented at the meeting, link it to prior efforts to answer these questions across the Wolbachia genus, and present the importance of these findings to the field of symbiosis. The topics covered in this review are based on the presentations at the conference.

## Abstract (precis)

Wolbachia pipientis is an alphaproteobacterial obligate intracellular microbe and arguably the most successful infection on our planet, colonizing 40% to 60% of insect species. Wolbachia spp.

## INTRODUCTION

## MOLECULAR MECHANISMS OF SYMBIOSIS

One major unifying theme emerged from the presentations, the attempt to discern the molecular mechanisms of symbiosis. The major phenotypes of Wolbachia spp. induced in the host include reproductive manipulations, but until now, we have not identified how the symbiont alters the host to produce these effects. Researchers have used models to try and make sense of the complex bidirectional incompatibility induced by the symbiont (1), have explored the influence of host and symbiont genotypes on the induced reproductive effects (2, 3), have studied the influence of environmental or ecological factors (4, 5), and have performed comparative genomics analyses (6) to try and identify the mechanism(s). A large increase in available genomes for analyses coupled to advances in our ability to detect proteins by mass spectrometry resulted in a major discovery in the field (7). A holy grail of arthropod Wolbachia research was found last year when two phage WO genes, *cifA* and *cifB*, were shown to mediate cytoplasmic incompatibility (8, 9). At the meeting, cytoplasmic incompatibility (CI) rescue was shown to be mediated by one of the same prophage WO genes which exist in an operon in the Wolbachia phage genome (10). Although this arrangement is reminiscent of toxin-antitoxin operons, Dylan Shropshire of the Seth Bordenstein lab suggested that this model may not be a clean representation for the induction of CI in Wolbachia spp. Shropshire showed both synthetic induction of CI, through transgenic expression of the toxin, as well as synthetic rescue, and presented a one-step model for the emergence of bidirectional CI. We now know that although both phage-encoded proteins are required for the induction of CI (9), overexpression of one alone can rescue the phenotype. Mark Hochstrasser presented work on the identification of these two phage-encoded proteins and the characterization of the toxin as a deubiquitinase (8), and Brittany Leigh of the Bordenstein lab presented work exploring how these proteins might be delivered to the host, perhaps by the phage itself. Furthering the mechanistic analysis of how CI is achieved, John Beckmann presented work using the Saccharomyces model system to identify eukaryotic targets for the CI toxin, which may include host proteins that are involved in nuclear import and chromosome structure. Mylene Weill brought this story to the field and presented how these CI loci have diversified across Wolbachia spp. infecting different Culex pipiens mosquitos (11). Strikingly, patterns of CI between these hosts are concordant with the similarities in the CI loci of their Wolbachia spp. For an excellent review of potential CI mechanisms and the evolution of the *cif* loci, see reference 12.

Regardless of the phenotype induced in the host, all Wolbachia strains share the need to colonize host cells and be efficiently maternally transmitted (13). In addition, to achieve their near-ubiquitous distribution, Wolbachia spp. must have mechanisms that promote transmission and maintenance in the cellular environments of different hosts. Wolbachia spp. encode and express a type IV secretion system, and it is likely that these symbionts modify the host environment via secreted effectors (13, 14). Researchers at the meeting presented work on requirements for Wolbachia colonization of hosts and how Wolbachia spp. modify host biology when in symbiosis. Many advances in the Wolbachia field have used the Drosophila melanogaster model system and leveraged what we know of fly biology and the ease of genetic tools to dissect the symbiosis (15–18). A significant body of work presented used the Drosophila model system and takes advantage of genetics in the host to identify pathways required for colonization. For example, Yolande Grobler of the Ruth Lehmann laboratory presented a high-throughput RNA interference (RNAi) screen to identify ribosome or translation initiation factors as important for Wolbachia colonization and found that host translation is inhibited by Wolbachia spp. (19). Grobler won a joint first-place presentation award for this exciting work that supports the observation that Wolbachia infection reduces translation in cell culture (20). Also using the D. melanogaster model, Horacio Frydman presented work showing that *wnt* signaling influences Wolbachia colonization of polar cells. Wolbachia spp. had previously been shown to require host microtubules for its localization within the developing oocyte (15) and to move along microtubule tracks. At the meeting, Shelbi Russell of the Bill Sullivan laboratory identified the competition between Wolbachia spp. and host germ line components during kinesin-mediated transport (21). A novel mechanism for Wolbachia titer control was presented by Teddy van Opstal of the Bordenstein lab, who used the Nasonia model to identify the taxon-restricted gene, *wds*, which maternally controls Wolbachia titer differences between closely related Nasonia species. These presentations identified new host components needed for Wolbachia colonization and emphasized the reliance of Wolbachia spp. on host cytoskeletal elements. One of the most exciting talks was that of Elves Duarte of the Luis Teixeira laboratory, who won a joint first-place award for his presentation. Duarte used ethyl methanesulfonate (EMS) to generate flies with overproliferative Wolbachia spp. He is taking a resequencing approach to identify potential polymorphisms or genetic ablations that lead to the overproliferative phenotype. He has observed that changes in the Octomom region are also present in these pathogenic lines. The region clearly serves to regulate Wolbachia titers, as this same group has correlated Octomom copy number with titer increases (22), but the mechanism has yet to be identified.

As Wolbachia spp. are intracellular bacteria, many in the field are interested in identifying how Wolbachia spp. survive in eukaryotic cells and how the symbiont alters host cell biology. Some of the most convincing and elegant studies have used microscopy to track Wolbachia localization and titer within important tissues, such as the reproductive tract (15, 16, 23, 24). Using this approach, Moises Camacho of the Laura Serbus laboratory presented microscopic quantification of Wolbachia sp. replication during oogenesis. Using 3-dimensional reconstruction of confocal microscopy images, Camacho’s data indicated exponential growth of Wolbachia across oogenesis and an approximation of possible Wolbachia replication rates within that tissue. Intuitively, one might expect that an intracellular infection would dramatically alter host biology, and indeed, Wolbachia spp. have been shown to alter host transcriptomics and proteomics (25, 26). At the meeting, several researchers presented work on modifications of the host environment by Wolbachia spp. For example, Denis Voronin showed that the glucose metabolism in parasitic filarial nematodes is likely mediated by host proteins on the surface of Wolbachia-containing vacuoles. Voronin’s results suggest an intimate metabolic association between Wolbachia spp. and their host, such that major host metabolic pathways may be used by the symbiont. Metabolic entanglements were also presented by Alexandra Grote of the Elodie Ghedin lab, where flux balance analysis suggests that many metabolic reactions in the Brugia filarial nematodes are provided by Wolbachia spp. These results would be further evidence of a mutualism between Wolbachia spp. and their filarial nematode hosts (27). Frederic Landmann presented work showing that in Brugia spp., Wolbachia spp. stimulate egg production and are essential to the germ line stem cell homeostasis. Mark Deehan of the Horacio Frydman lab showed that rapamycin treatment of flies alters Wolbachia titer and therefore that an increase in autophagy decreases Wolbachia density suggesting. This result echoes previous work by Denis Voronin suggesting that Wolbachia populations are regulated by host autophagy. Towards the identification of a mechanism mediating these types of interactions, Irene Newton presented work on candidate proteins secreted by Wolbachia spp. via the type IV secretion system (T4SS). Although all Wolbachia strains sequenced thus far contain the genes encoding the T4SS machinery, the proteins secreted by these systems (the effectors) have not been well characterized across the genus (28). In the host Drosophila melanogaster, the candidate effectors are coregulated with the T4SS, and one secreted substrate, WalE1, interacts with actin and actin-regulatory proteins (28, 29). Amelia Lindsey of the Irene Newton laboratory presented work showing that Wolbachia spp. alter the expression of its T4SS and effectors in response to host-derived signals.

## USING WOLBACHIA SPP. TO LIMIT VECTORED DISEASES

The recent claim to fame of Wolbachia spp. is their ability to inhibit pathogen replication (for a review, see reference 30), and a large body of research was presented at the meeting, further characterizing this phenomenon and determining at the mechanism. For example, Beth McGraw used experimental evolution to identify genetic variance in both Aedes aegypti and Wolbachia sp. strain *w*Mel with regard to pathogen blocking through selection on that trait. Interestingly, Wolbachia density, long thought to be correlated with extent of pathogen blocking across host systems (31), did not explain the increase in protection. With regards to what mechanism might explain RNA virus inhibition mediated by the symbiont, Manabu Ote suggested that RNAs might be generally targets for Wolbachia-mediated phenotypes, explaining pathogen blocking as a side effect of host-Wolbachia interaction, while Tamanash Bhattacharya of the Irene Newton laboratory presented data suggesting that Wolbachia spp. alter the expression of a host methyltransferase to epigenetically modify virus RNA genomes and won a presentation award (32). The session ended with sobering words from two presentations, in which both Jason Rasgon and Heather Flores reminded the attendees that the pathogen-blocking phenotype is more complex and dependent on host-symbiont-pathogen combination (33, 34). Finally, Wolbachia spp. are also known to inhibit other pathogens, beyond viruses, and Fabio Gomes, who won a presentation award, described Anopheles mosquitoes containing Wolbachia spp. that reduce malaria transmission.

Another way to limit pathogen spread is to control vector populations, and Wolbachia spp. have a long history as biocontrol agents. Several potential strategies exist for the use of Wolbachia spp. in arthropod-based disease control (35, 36). These can be divided into two broad categories, Wolbachia population reduction or Wolbachia population replacement. The first general strategy is to reduce insect populations by using CI to produce males which, when released in the population, mate to indigenous females, resulting in defective embryogenesis. This is a form of an incompatible insect technique (IIT) using Wolbachia spp., an offshoot of an older sterile insect technique (SIT) (36) in which repeated introductions of sterile males (created by irradiation or chemical sterilization) are released to mate and reduce population size. IIT relies on CI, in which females are effectively sterilized when they mate with males harboring no or an incompatible Wolbachia strain. Since male mosquitoes do not feed on blood and thus do not transmit disease, extensive or repetitive release of male mosquitoes is not a health or nuisance issue. In this light, presentations by Zhiyong Xi and Kaycie Hopkins described the creation of “mosquito factories” wherein sterile males are bred for release to reduce mosquito populations and thus the incidence of human arthropod mosquito-borne viral diseases. The second strategy, populational replacement, is not designed to reduce population size but uses CI to replace females in the population with Wolbachia-infected females that have the ability to block or reduce pathogen transmission. CI can thus create a “populational sweep” to enable the introduced Wolbachia spp. to obtain high infection frequency in the population.

## WOLBACHIA SPP. IN FILARIAL NEMATODES

Most human filarial nematode parasites are hosts for Wolbachia spp. Intracellular bacteria were first detected in filarial nematode tissues by electron microscopy (37, 38) and later identified as belonging to Wolbachia by molecular analyses (39). While not all filarial nematodes have Wolbachia spp., when present, they are mutualists, required for normal worm development, fertility, and survival. Due to their obligate nature in those filarial parasites, Wolbachia spp. have been a novel drug discovery target using a variety of approaches, including diversity and focused library screening and the use of genomic sequence analysis to identify gene products required for maintenance of the symbiotic relationship (40, 41). Current antifilarial drugs only affect the microfilarial stages and thus require repeated mass drug administrations to eliminate them as they are continually produced by the filarial nematode adults. *In vitro* and *in vivo* anti-Wolbachia antibiotic treatments have been shown to have adulticidal activity, a long-sought goal of filarial parasite drug discovery. A goal of anti-Wolbachia drug discovery has also been to reduce the time needed for administration (for example, doxycycline requires 4 to 6 weeks of treatments) and remove any antibiotic counterindications for women of child-bearing age and for children under the age of 6. At the meeting, Mark Taylor highlighted the latest outputs from the anti-Wolbachia drug screening program A-WOL (https://awol.lstmed.ac.uk/) aimed to discover and develop new curative anti-Wolbachia drugs and regimens for onchocerciasis and lymphatic filariasis (42). A major goal of the project, a macrofilaricide course of treatment of 7 days or less, has now been achieved with combination drug therapy (43), and with new candidate drug monotherapy with TylaMac and AW1066 entering phase I trials and further preclinical testing.

## A LARGER SYMBIOSIS FRAMEWORK INCLUDING SPIROPLASMA SPP. AND OTHER SYMBIOSES

The Wolbachia meetings have always been home to researchers on reproductive manipulators outside the genus, such as for Spiroplasma. Toshiyuki Harumoto of the Bruno Lemaitre lab presented his discovery of a male-killing toxin in Spiroplasma poulsonii, which they named *spaid* (44). This “androcidin,” predicted since the early 1970s, is located on a plasmid and encodes both an ankyrin domain and an ovarian tumor (OTU) domain. Transgenic expression of the entire gene recapitulated male killing. A potential mechanism for Wolbachia male killing was also presented by Jessie Perlmutter of the Bordenstein laboratory; yet, another prophage gene, named *wmk*, kills males preferentially upon transgenic expression. Another mechanism of symbiosis in Spiroplasma spp. was presented by Steve Perlman. Spiroplasma spp. confer protection in Drosophila spp. against both parasitic nematodes and wasps, and he identified a diverse repertoire of ribosome-inactivating toxins (RIPs) that target ribosomes of parasites developing in the host (45, 46). He further showed that these toxins are common in protective symbioses and that they cleave 28S rRNA of the invading parasite. The meeting also included presentations from Maki Inoue and Daisuke Kageyama on male killing and viruses, such as partitiviruses, that produce separate capsids for each genomic segment.

Just as symbionts alter host cell biology, the external environmental conditions can shape endosymbiont dynamics inside the host, and as a result, a different phenotype can be observed. The prevalence and penetrance of symbioses, therefore, can vary across years and seasons. For example, Martha Hunter discussed variation in infection frequency of symbiont Rickettsia spp. in the sweet potato whitefly Bemisia tabaci over 16 years (2000 to 2016). Her group saw symbiont frequency climb from 1% to 97% from 2000 to 2006 (47), stay high through 2011 (48), and then drop to 36% in 2017. A similar fluctuation in the prevalence of Hamiltonella defensa across 6 months was observed in the pea aphid by Jacob Russell. Temperature, altitude, and host plants determined the infection dynamics of Wolbachia, Cardinium, and Spiroplasma spp. in spider mites (Tetranychus truncatus), as investigated by Xiao-Yue Hong. He found that Wolbachia infection was more prevalent in the geographical area with high mean temperature, but hosts infected with Cardinium and Spiroplasma were likely to be found at higher altitude. Temperature also took center stage in a study by Amy Truitt, who showed that Wolbachia infection modified thermal preference in Drosophila melanogaster. Flies infected with the bacterium preferred cooler temperatures than uninfected flies, and the influence was strain dependent (49). Jennifer Morrow described the presence of primary and secondary symbionts in psyllid species (50), suggesting strict vertical transmission of a minimal endosymbiont “core” and yet divergence of symbionts and gene families to maintain functional metabolic integrity. Ben Makepeace followed “*Candidatus* Midichloria” in European tick populations suggesting widespread horizontal transmission (51). Finally, Takema Fukatsu presented the discovery that across some cicada species, the bacterial symbiont Candidatus *Hodgkinia* has been replaced by Ophiocordyceps fungi (52). This fungus, which normally parasitizes cicadas, has been recruited as a mutualistic symbiont in some lineages.

## WOLBACHIA GENOMICS AND TRANSCRIPTOMICS

Because Wolbachia spp. cannot be cultured outside host cells, much of what we know about Wolbachia spp. has come from genomic and transcriptomic sequencing studies. However, how does one target and sequence the genetic material from an intracellular symbiont? At the 2018 meeting, several new bioinformatics and wet lab approaches were presented to facilitate the sequencing process, including approaches by Mark Gasser of the Julie Dunning Hotopp laboratory, who presented results using MinION long-read sequencing for Wolbachia spp., a strategy that allowed him to assemble the entire prophage region easily with the long reads. Emilie Lefoulon from the Slatko laboratory presented an enrichment strategy for a supergroup J Wolbachia genome and also turned heads as she described the smallest Wolbachia genome yet, at 864,015 bp, and missing the gene encoding the cell division protein FtsZ. Genomics tools were also presented by Julie Dunning Hotopp, who used stage-specific transcriptome sequencing to show that, unlike Wolbachia sp. *w*Mel in Drosophila spp., Wolbachia sp. strain *w*Bm in the nematode Brugia malayi does not regulate its gene expression based on host developmental stage. Wolbachia genomic fragments are known to have integrated into host genomes over evolutionary timescales (lateral gene transfer [LGT] events) (53, 54), and two presentations at the meeting focused on these LGTs. Robin Bromley of the Dunning Hotopp lab presented new software (LGTSeek) based on short-read junctions to assist in finding LGTs within insect genomic data sets (http://www.igs.umaryland.edu/labs/lgthgt/analysis/lgt-seek/), while Alistair Darby presented his data on LGTs within Aedes albopictus. These LGTs can have a significant impact on host biology, as seen in pillbugs (55) and, more recently it seems, in booklice (56).

## EVOLUTION AND SPREAD OF WOLBACHIA SPP.

Because Wolbachia spp. often manipulate host reproduction, benefiting females by increasing their relative fitness in populations, the microbe can have significant consequences on host evolution (57, 58). Wolbachia infections can enter new populations, spread, and persist within them by the reproductive manipulations for which it is famous (59, 60), although some strains seem to induce no phenotypic effect at all in their hosts (61, 62). In related work, Guilherme Baião used transcriptome sequencing to identify Wolbachia-regulated transcripts in Drosophila paulistorum, detailing the influence of Wolbachia on pre- and postmating isolation mechanisms, possibly contributing to host speciation. Brandon Cooper presented comparative population genomics of Wolbachia spp. within the hybridizing Drosophila yakuba clade in West Africa, where infection frequencies vary across time and space. Cooper observed that some host loci yet to be identified modulate cytoplasmic incompatibility in Drosophila teissieri. Michael Turelli, in collaboration with Brandon Cooper, Will Conner, Ary Hoffman, and colleagues, discussed the predominant modes of Wolbachia acquisition, including the observation that Drosophila hosts diverged up to 50 million years ago have Wolbachia spp. that diverged only a few thousand years ago (63). These results suggest that Wolbachia spp. rapidly invade new host species through either introgression or horizontal acquisition.

The evolution of the Wolbachia genus, and the placement of different clades within the genus (known as supergroups), has been a topic of some debate (64–69). At the meeting, Michael Gerth, who won a presentation award, cautioned that the use of multilocus sequence typing (MLST) primer sets may not be an accurate determiner of species identification and that analysis using genome sequencing, if sequences were available, would be more accurate method. He also presented a provocative phylogeny that rooted the Wolbachia tree at supergroup B, suggesting that nematode association was a secondary adaptation. The Wolbachia 2018 meeting highlighted exciting new developments in the field from functional genomics to biochemistry and genetics. Each meeting ends with a brief topic for attendee discussion, and this year, the topic revolved around symbiont evolution and Wolbachia supergroup/species naming (for more background on this debate, see references 70 and 71). Based on that discussion, a working group was formed, headed by Julie Dunning Hotopp, to create a viable nomenclature system for Wolbachia systematics (72).

## CONCLUSIONS AND FUTURE DIRECTIONS FOR WOLBACHIA RESEARCH

Wolbachia research transverses biology. The Wolbachia 2018 meeting highlighted exciting new developments in the field, including functional genomics, biochemistry, development, cell biology, genetics, and population ecology. Over 100 participants from 20 countries attended, and there were over 60 oral presentations and 20 posters covering a wide range of topics, including species-level identification, development, cell biology, genomics, ecology, and evolution. The conference was funded by grants from the National Science Foundation (NSF), Burroughs Wellcome Fund, Pacific Biosciences, Mini-One, EmbiTec, and New England BioLabs. A complete list of speakers, the abstract book, and topics can be found on the website (https://wolbachia2018.org/). At this year’s Wolbachia meeting, the field seemed to have come of age. Major discoveries were presented, including the identification of toxins secreted by symbionts to alter host reproduction and the host pathways and cell biology required for maintenance of the infection, but major questions remain in the field. For example, the host targets of the *cif*-encoded proteins have not been identified, and the mechanism by which these toxins induce CI is not yet known. Are the mechanisms of reproductive manipulation conserved across bacterial symbionts of insects? The presence of a deubiquitylase domain in toxins found across the *Rickettsiales* clade, in Wolbachia spp., and in Spiroplasma spp. might suggest this (73). Outstanding challenges in the field include the inability to culture Wolbachia spp. *ex vivo* and genetically manipulate the microbe, but exciting new approaches make linking genotype to phenotype more likely in the future. For example, the ability to generate new Wolbachia-infected cell lines has facilitated the study of the microbe and its use in vector control (74). Major questions remain with regard to the evolution of the genus, such as which came first, the insect-associated clades or the nematode associations? With more genomes coming online every month, perhaps this long-standing question will finally be answered. The next Wolbachia meeting will be held in Crete in 2020, and the community looks forward to advances and answers to some of these questions.
